# Long-term consequences of developmental vascular defects on retinal vessel homeostasis and function in a mouse model of Norrie disease

**DOI:** 10.1371/journal.pone.0178753

**Published:** 2017-06-02

**Authors:** Susanne C. Beck, Yuxi Feng, Vithiyanjali Sothilingam, Marina Garcia Garrido, Naoyuki Tanimoto, Niyazi Acar, Shenliang Shan, Britta Seebauer, Wolfgang Berger, Hans-Peter Hammes, Mathias W. Seeliger

**Affiliations:** 1 Division of Ocular Neurodegeneration, Institute for Ophthalmic Research, Centre for Ophthalmology, Tuebingen, Germany; 2 Institute of Experimental and Clinical Pharmacology and Toxicology, Medical Faculty Mannheim, University of Heidelberg, Mannheim, Germany; 3 Institute of Medical Molecular Genetics, University of Zurich, Zurich, Switzerland; 4 Center for Integrative Human Physiology (ZIHP), University of Zurich, Zurich, Switzerland; 5 Neuroscience Center Zurich (ZNZ), University and ETH Zurich, Zurich, Switzerland; 6 5th Medical Department, Medical Faculty Mannheim, University of Heidelberg, Mannheim, Germany; University of Florida, UNITED STATES

## Abstract

Loss of Norrin signalling due to mutations in the Norrie disease pseudoglioma gene causes severe vascular defects in the retina, leading to visual impairment and ultimately blindness. While the emphasis of experimental work so far was on the developmental period, we focus here on disease mechanisms that induce progression into severe adult disease. The goal of this study was the comprehensive analysis of the long-term effects of the absence of Norrin on vascular homeostasis and retinal function. In a mouse model of Norrie disease retinal vascular morphology and integrity were studied by means of *in vivo* angiography; the vascular constituents were assessed in detailed histological analyses using quantitative retinal morphometry. Finally, electroretinographic analyses were performed to assess the retinal function in adult Norrin deficient animals. We could show that the primary developmental defects not only persisted but developed into further vascular abnormalities and microangiopathies. In particular, the overall vessel homeostasis, the vascular integrity, and also the cellular constituents of the vascular wall were affected in the adult Norrin deficient retina. Moreover, functional analyses indicated to persistent hypoxia in the neural retina which was suggested as one of the major driving forces of disease progression. In summary, our data provide evidence that the key to adult Norrie disease are ongoing vascular modifications, driven by the persistent hypoxic conditions, which are ineffective to compensate for the primary Norrin-dependent defects.

## Introduction

Correct retinal vascularization together with retinal vessel homeostasis are essential for normal ocular function. An imbalance in these processes contributes to numerous sight threatening ocular diseases. The causes, however, are multi-faceted and encompass developmental disturbances like in retinopathy of prematurity (ROP) [1], as well as metabolical conditions that occur in diabetic retinopathy [2] but also genetic alterations that account for inherited developmental disorders like Norrie disease.

Norrie disease is caused by mutations affecting the *NDP* (Norrie disease pseudoglioma) gene that is encoding for the Norrie protein [3] and characterized by progressive deafness, mental retardation, but also congenital blindness due to malformations in the retina [4]. The typical clinical signs in the eye are bilateral retinal degeneration and extensive vitreous membranes. Mutations in the *NDP* gene also account for a variety of other familial and sporadic diseases, including exudative vitreoretinopathy [5], advanced retinopathy of prematurity [6], and Coats disease [7]. In 1996, a mouse model for Norrie disease was generated by homologous recombination in embryonic stem cells (*Ndph*^*y/-*^ mouse) [8]. In these mice, retinal degeneration and vitreoretinal membranes develop similar to those observed in Norrie disease subjects [9]. Another similarity between patients with mutations in the *NDP* gene and the mouse model of Norrie disease is that the retinal changes in *Ndph*^*y/-*^ mice are accompanied by prominent defects within the retinal vasculature and by persistent hyaloid vessels in the vitreous [10, 11]. In the study published by Richter et al. [11] different kinds of defects were observed within the retinal vasculature ranging from a lack of vessels in the outer layers to an increase in the number of vessels in the inner retinal layers, some of the capillaries being fenestrated in the inner retina. At that time, the abnormal retinal function of *Ndph*^*y/-*^ mice was attributed to retinoschisis-like alterations of the retina rather than to the abnormal vascularization [9].

By characterizing the time course of Norrie disease in terms of retinal and hyaloid vasculature and by identifying the molecular angiogenic pathways involved, we could provide direct evidence for a crucial role of Norrin in hyaloid vessel regression and in sprouting angiogenesis during the formation of the deep retinal capillary networks [12, 13]. These studies performed during early life in animals aged from 5 days to 21 days demonstrated that the clinical course of Norrie disease is divided into two phases. In an early period taking place until postnatal day 15, the lack of Norrin leads to the abnormal growth of deep retinal capillaries due to defects in sprouting angiogenesis. From postnatal day 15, the lack of well-developed retinal vasculature results in a severe retinal hypoxia that is characterized by an upregulation of HIF1α and VEGFα proteins and a negative electroretinographic response [12].

The experimental work published so far focused on the developmental aspects of the Norrie syndrome [12–15] but only little is known about later stages of the disease. Given the fact that correct vascularization and vascular homeostasis are crucial for ocular function the scope of this study was to analyse the long-term consequences of the defective vasculature in a mouse model of Norrie disease. In this study, we were particularly interested in potential adaptive and/or compensatory mechanisms in the mature retina. To address this question we studied the overall appearance and integrity of the retinal as well as intraocular vasculature *in vivo*. The morphology of the mature retinal vasculature was analysed ex vivo and morphometric characteristics of the vascular constituents were quantified. Moreover, we investigated the effect of long-term Norrin deficiency on the functional properties of the mature neural retina. In summary, we observed a close interplay between the primary developmental defects and secondary hypoxia-driven alterations that affected not only the constituents of the retinal vasculature but also the vessel integrity and the functional properties of the mature Norrin depleted retina.

## Materials and methods

### Ethics statement

All procedures were performed in accordance with the local ethics committee (Regierungspraesidium Tuebingen), German laws governing the use of experimental animals, and the ARVO statement for the use of animals in ophthalmic and visual research. The Institute of Animal Welfare and the Veterinary Office at the University of Tuebingen insures compliance with all applicable regulations for the use of animals. All examinations are approved by The Institute of Animal Welfare and the Veterinary Office at the University of Tuebingen and the Regierungspraesidium Tuebingen.

### Animals

The *Ndp*^*y/-*^ mouse line was generated by Berger et al. as described previously [8]. The mutation is kept on a C57BL/6J background. Genotyping was performed by PCR analysis of ear DNA [8].

### *In vivo* angiography

Scanning-Laser Ophthalmoscopy (SLO) was performed after the functional assessment of the animals by means of Electroretinography (ERG). Retinal structures of the anesthetized animals were visualized with an HRA 2 (Heidelberg Engineering, Heidelberg, Germany) according to a previously published method [16]. Briefly, the HRA 2 system features lasers in the short (visible) wavelength range (488 nm and 514 nm), and also in the long (infrared) wavelength range (785 nm and 815 nm). To follow the vascular changes in the eyes of 2 months old *Ndph*^*y/-*^ mice *in vivo*, we used fluorescein (FL) with the argon blue laser (488 nm; barrier, 500 nm) and indocyanine green (ICG) with the infrared laser (795 nm; barrier 800 nm). Analysis of the anterior part of the eye ranging from the lens down to the retinal surface was also accomplished by means of SLO angiography to analyse the still existing remnants of the fetal hyaloid vasculature.

### Retinal whole mount staining

For collection of the eyes the mice were euthanised by CO_2_ inhalation. After enucleation the eyes were fixed in 4% PFA for 2h, and retinas were dissected and washed three times with PBS for 1 hour, then incubated in permeabilisation buffer (1% BSA, 0.5% Triton-100 in PBS) for 1 hour at room temperature. Retinas were incubated in Lectin-FITC diluted in permeabilisation buffer overnight at 4°C. After washing three times for 1 hour again with PBS, the samples were covered. Photographs were taken with a microscope connected to a video camera (Leica, Wetzlar, Germany).

### Analysis of retinal vasculature in eye cryosections

Retinal cryosections (12-mm thick) were cut and collected on slides. Sections were fixed in 4% formalin, then, incubated in a blocking solution and permeabilisation buffer for one hour at room temperature. The sections were incubated with Lectin-TRITC diluted in the blocking solution at 4°C overnight. Slides were washed with PBS again and mounted with anti-fade medium (10% Mowiol 4–88 (vol/vol; Calbiochem, San Diego, CA, in 100 mM Tris (pH 8.5), 25% glycerol (wt/vol) and 0.1% 1,4-diazabicyclo [2.2.2] octane (DABCO).

### Retinal digest preparation and quantitative morphometry

Eyes were obtained following the SLO imaging step. Retinal vascular preparations were performed using a trypsin digestion technique as previously described [17]. Briefly, the retinas were fixed in 4% formalin for 2h and subsequently incubated in 3% trypsin solution resolved in 0.2 mol/L Tris buffer (pH 7.4) for 120 min. For analysis of intraretinal vascular morphometry, the vessels above the inner limiting membrane were carefully removed. Subsequently, the retinal digest preparations were carefully washed with aqua bidest and flat mounted on slides. Finally, the samples were stained using periodic-acid Schiff reagent (PAS). The numbers of aneurysms were counted in PAS-stained retinal digest preparations of two-month-old wildtype (n = 5) and *Ndp*^*y/-*^ mice (n = 5). The diameters of retinal arterioles, venules, capillaries and microangiopathies were measured using quantitative retinal image analysis (Analysis Pro System; Olympus Opticals, Hamburg, Germany).

### Functional studies based on ERG

ERGs were recorded binocularly from two months old mice according to previously described procedures [18]. Mice were anaesthetized using a combination of Ketamine (66.7 mg/kg body weight) and Xylazine (11.7 mg/kg body weight). Their pupils were dilated and single flash ERG responses were obtained under scotopic (dark adapted overnight) and photopic (light adapted with a background illumination of 30 cd/m2 starting 10 min before recording) conditions. Single white-flash stimuli ranged from -4 to 1.5 log cd*s/m2 under scotopic and from -2 to 1.5 log cd*s/m2 under photopic conditions. Ten responses were averaged with inter-stimulus intervals of 5 s (for -4 to -0.5 log cd*s/m2) or 17 s (for 0 to 1.5 log cd*s/m2).

### Statistical analyses

Data are expressed as mean ± standard deviation (SD). The data were analyzed using the GraphPad Prism software (GraphPad Software, La Jolla) or SAS release 9.3 (SAS institute Inc., Cary, North Carolina, USA). Unpaired T-test or Analysis of Variance (ANOVA) for quantification of intraretinal vessel parameters was used. A value of p < 0.05 was considered statistically significant.

## Results

### Developmental vascular defects persist in the mature *Ndph*^*y/-*^ retina

**Fig 1:**
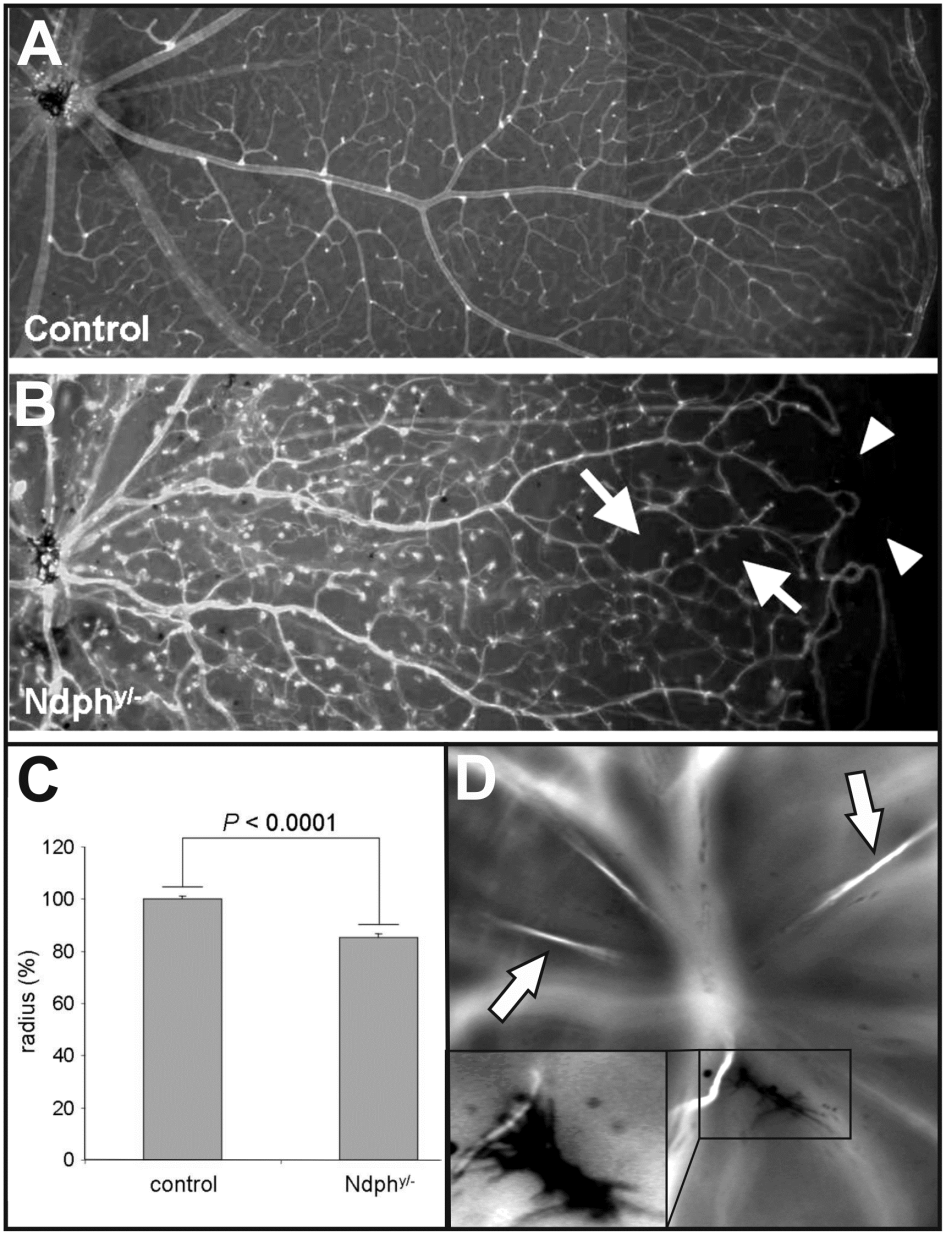
Early developmental defects persist in 2-months-old *Ndph*^*y*/-^mice. Lectin-FITC-stained retinal whole mount preparations of age matched control (A) and Norrin deficient mice (B) detected reduced outgrowth of the retinal primary plexus (arrowheads), avascular zones and large periarteriolar capillary free zones (arrows). (C) Morphometric quantitation of vessel outgrowth towards the periphery of the *Ndph*^*y/-*^ mice and age-matched controls at 2 months of age (n = 3) resulted in a significantly reduced percentage coverage of the retina by superficial capillary network in *Ndph*^*y/-*^ mice (*P* < 0.0001, unpaired *t* test). Data are mean ± SD of radius (%) which is radius (vasculature) / radius (retina), of the retinal arteries in the wild-type and *Ndph*^*y/-*^ mice. (D) The delayed regression of the primary vitreous resulted in still functional hyaloid vessels (arrows) as well as remaining condensed tissue-like material within the vitreous body (inset) resembling the vitreoretinal membranes in patients.

Norrin dependent developmental defects in the retinal vasculature of young animals are characterized by the lack of deeper retinal capillaries and an avascular retinal periphery [12, 13]. In this study we observed that the retina was still not completely vascularized in the old *Ndph*^*y/-*^ mice at two months of age. The reduced outgrowth of the superficial capillary network toward the periphery was evident in Lectin-FITC-stained retinal whole mount preparations (Fig 1B). In contrast to the retinal vasculature of age-matched control animals (Fig 1A), in mutant mice the arteries of the primary plexus did not reach the retinal margin resulting in complete avascular areas in far periphery (Fig 1B, arrowheads). Furthermore, the retinal periphery of Npdh^y/-^ mice was also characterized by large periarteriolar capillary-free zones (Fig 1B, arrows). The morphometric quantitation of vessel outgrowth toward the periphery of the *Ndph*^*y/-*^ mice and age-matched controls revealed that the superficial vessels covered 85% ± 1.4% of the entire retina in mutant mice whereas the percentage coverage was maximal in control animals (Fig 1C). Remarkably, the percentage coverage of retinal superficial vessels in 2-months-old *Ndph*^*y/-*^ mice was higher than that measured at postnatal day 21 (75% of the retinal surface) [12], suggesting that angiogenesis continued in *Ndph*^*y/-*^ mice after postnatal day 21.

During development, the hyaloid vasculature nourishes the avascular regions of the retina. In close correlation with the growth of retinal vasculature these fetal vessels regress. In wild type mice the hyaloid vasculature has completely regressed between p14 and p21 [19]. In adult *Ndph*^*y/-*^ mice, however, several still functional hyaloid vessels could be detected in *in vivo* angiography (Fig 1D, arrows). As it is shown in Fig 1B, the retina was still not completely vascularized. Therefore, the functional preservation of the fetal vasculature could be a mechanism to compensate for the avascular regions in the periphery. Furthermore, the irregular and delayed regression of the primary vitreous resulted in condensed tissue-like structures attached to an open hyaloid vessel (Fig 1D, inset) within the vitreous body. This morphological characteristic closely resembled the vitreoretinal membranes that develop also in patients carrying mutations in the *NDP* gene [4].

### Remodelling of the entire primary superficial capillaries into microangiopathies

**Fig 2:**
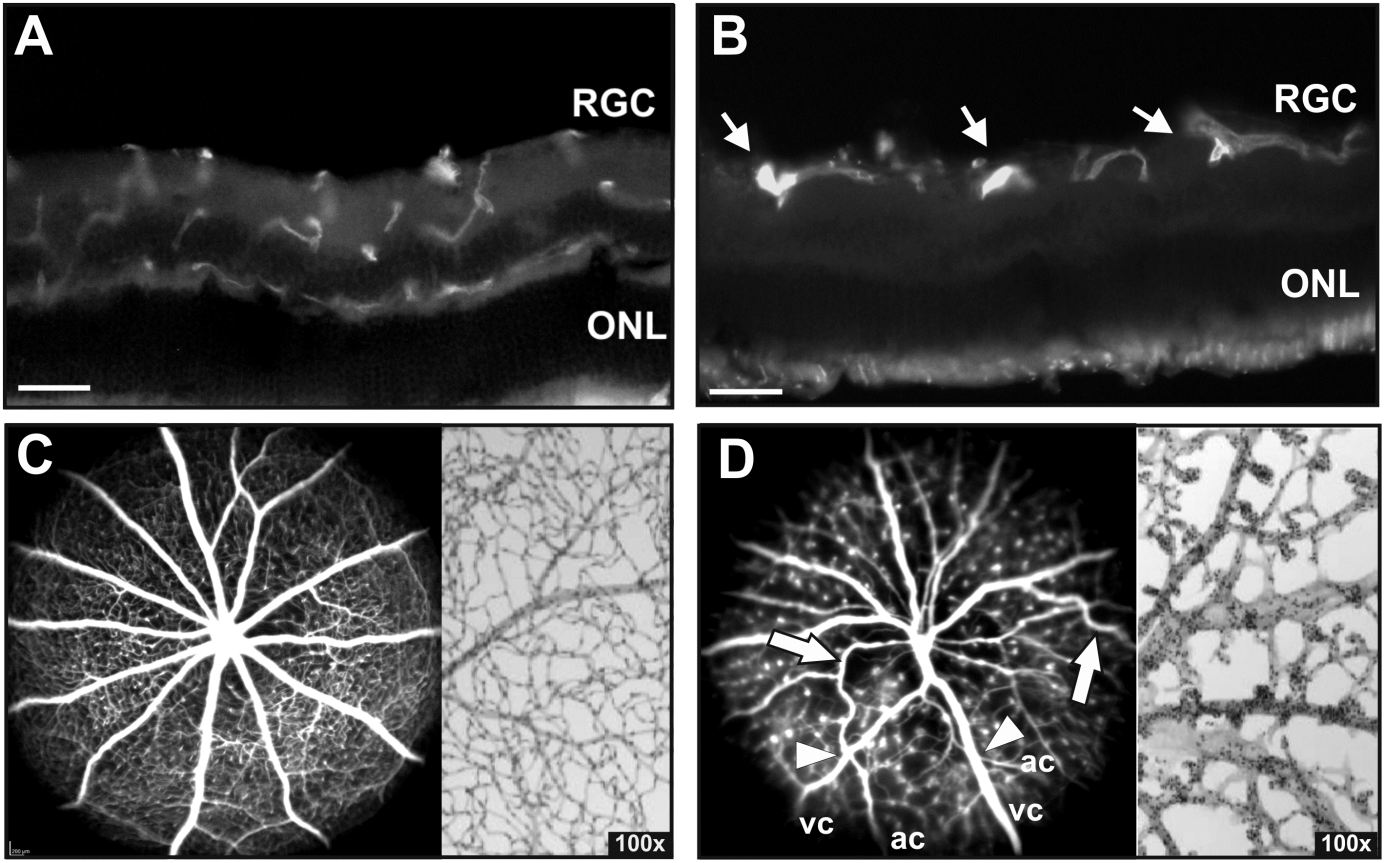
Defects in the superficial retinal vasculature. (A, B) Retinal cryosections stained with Lectin-TRITC. In contrast to control animals (A), the lectin-positive cells are confined at the level of nerve fiber layer in 2-months-old *Ndph*^*y/-*^ mice indicating no development of retinal deep capillaries (B). Highly fluorescent lectin-positive dots that correspond to cellular aggregations were observed in *Ndph*^*y/-*^ mice (B, arrows). Analysis of the retinal vasculature and capillary network by *in vivo* angiography with FL (C, left) and PAS stained retinal digest preparations (C, right) in control animals. *In vivo* angiography with ICG (D, left) and ex vivo retinal digest preparations (D, right) revealed severe vascular alterations in the retina of 2 months old *Ndph*^*y/-*^ mice. The deep capillary network has still not formed resulting in a complete transformation of the superficial capillaries into microaneurysm-like lesions presenting as small fluorescent dots in ICG angiography (D, left) and knob-like structures in retinal digest preparations (D, right). Moreover, retinal arteries presented a tortuous appearance with large bends indicating an incorrect attachment to the underlying tissue (D, arrows). Additionally, vessel crossings could be observed (D, arrowhead, ac, arterial character; vc, venous character). Scale bar, 50μm.

In the Norrin depleted retina the deeper capillary network fails to develop. In adult *Ndph*^*y/-*^ mice lectin staining of eye cryosections revealed that the distribution of retinal blood vessels within the inner retinal layers was still abnormal (Fig 2B). In mutant animals, the lectin-positive cells were confined to a small area in the inner retina at the level of the nerve fiber layer. These cells correspond to the retinal primary vasculature, thus confirming that the intermediate and the deep capillary beds had still not formed in the inner and the outer plexiform layers. Moreover, in the retina of 2-months-old *Ndph*^*y/-*^ mice large highly fluorescent lectin-positive dots (Fig 2B, arrows) were observed, whereas the lectin-positive cells in the retina of wild-type control animals were widely distributed within the inner retinal layers as they match with the normally developed superficial, intermediate and deep capillary layer (Fig 2A). In *in vivo* SLO angiography these hyper fluorescent dots were correlated to microaneurysm-like lesions (Fig 2D, left). However, in contrast to p15 and p21, where remnant areas of a superficial capillary network could still be detected [12], in the retina of 2-months-old adult *Ndph*^*y/-*^ mice the complete capillary network had been transformed to drum stick-like microangiopathies (Fig 2D, *in vivo* angiography (left), retinal whole mount digest preparation (right)) indicating a continuous vascular remodelling between p21 and 2 months of age.

Moreover, SLO angiography revealed that the Norrin dependent irregularities are not only confined to the capillaries but also affected the large retinal vessels. In adult *Ndph*^*y/-*^ mice retinal arteries presented a tortuous appearance with large bends (Fig 2D, arrows). This anatomic particularity may correspond to the long-term remodelling of the retinal arteries that were lifted over the network holes, because still after 2 months of age, they were not correctly attached to the underlying tissue, as observed by Luhmann et al. [12] at day 14. Additionally, extensive vessel crossing was still present in adult *Ndph*^*y/-*^ retinas corresponding to the observations in younger animals between P7 and P27 [13]. Vessels of venous character (vc) crossed vessels morphologically resembling arteries (ac) (Fig 2D, arrowheads).

**Fig 3:**
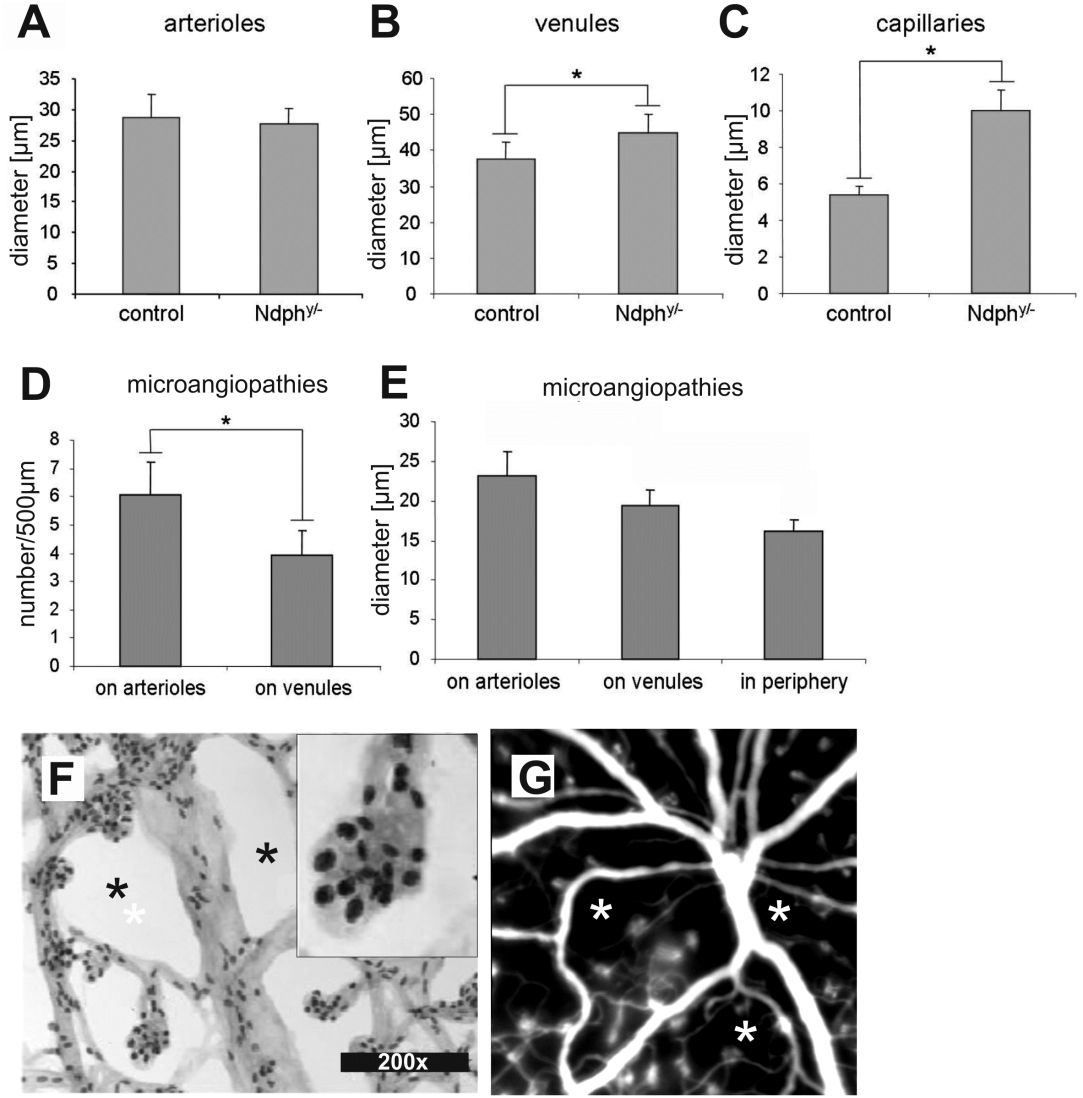
Detailed analysis and quantification of retinal morphometric characteristics. (A-E) Quantitative analysis of the vascular constituents in PAS stained retinal digest preparations. *Ndph*^*y/-*^ mice (n = 5) exhibited no difference in the diameter of their retinal arterioles (A) when compared to age-matched controls whereas those of venules (B) and capillaries (C) were increased by 20% and by 85%, respectively (P < 0.05 and P < 0.001 respectively, unpaired t-test). In *Ndph*^*y/-*^ mice, the microaneurysms (D) were more abundant on arterioles than on venules (P < 0.001, unpaired t-test) and (E) they were significantly wider in the center than in far periphery (P < 0.01, ANOVA). (F) Magnifications of retinal digest preparations confirmed the cellular aggregations in the microaneurysm-like structures as suggested from lectin-stained retinal whole mounts (Fig 2B). According to shape and staining mainly pericytes could be identified as constituents of these clusters (F, inset). In contrast to the evenly distributed capillary network of wild-type animals (Fig 2C) several capillary free zones were not only detected in the far periphery (Fig 1B) but also in the central part of the retina (F, asterisks) and even close to the optical disc (G, asterisks).

In the normal retina (Fig 2C, angiography (left) and retinal digest preparation (right)) and in the young *Ndph*^*y/-*^ retina [12], both arteries and veins, did not differ very much in size. However, in the adult Norrin deficient retina, the large retinal vessels appeared considerably altered in their dimensions. The veins appeared dilated and the arteries were quite smaller in diameter in *in vivo* angiography (Fig 2D, left) which very likely could be considered as a response to the altered blood flow due to the missing intermediate and deep capillary layers. Detailed morphometric analyses of retinal digest preparations revealed a considerable enlargement of the venules (Fig 3B) as well as the capillaries (Fig 3C) whereas the arterioles appeared to be unchanged (Fig 3A).

**Fig 4:**
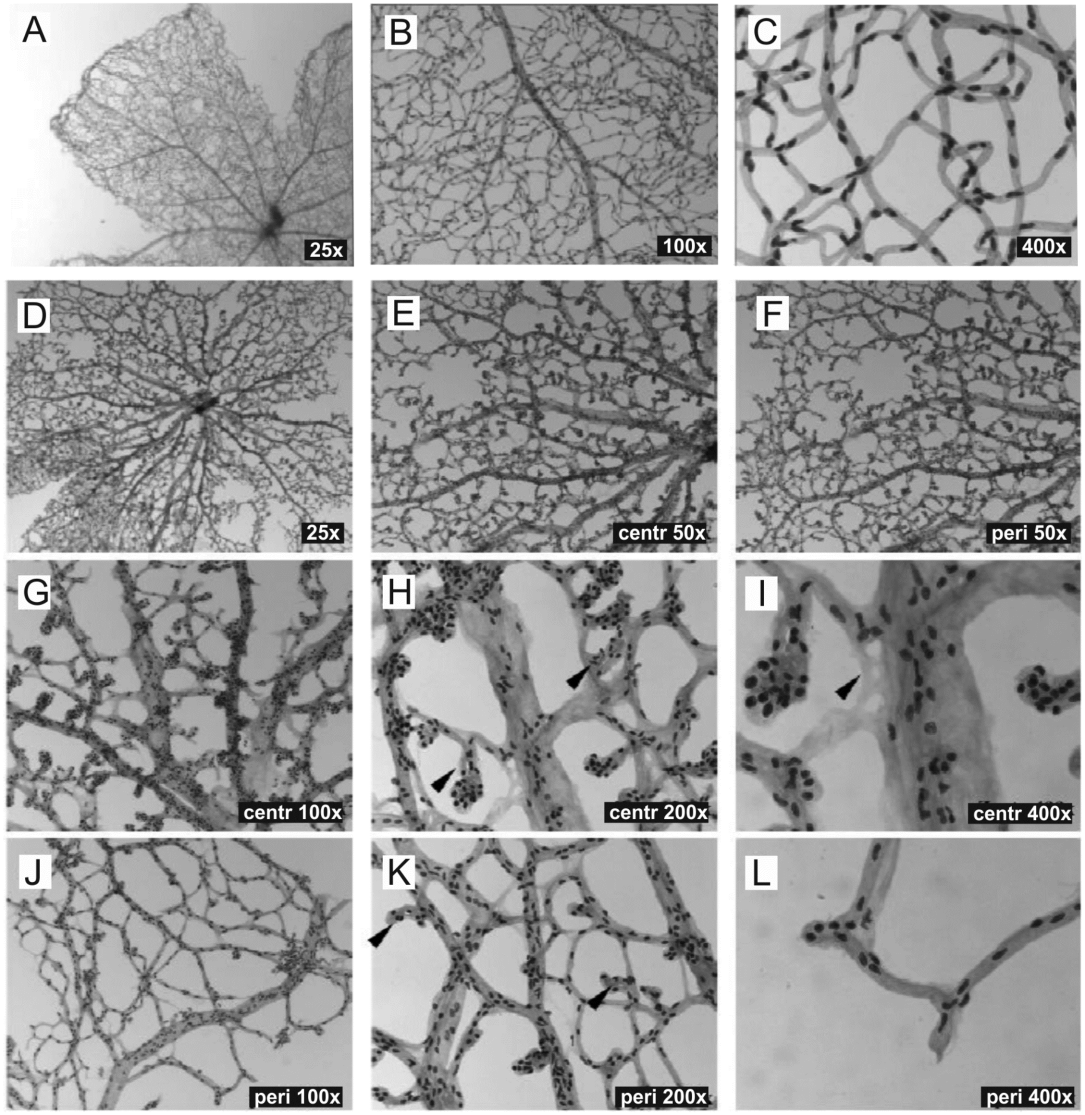
Detailed analysis of the retinal vasculature in PAS stained digest preparations. (A-C) Wild-type mice at 2 months of age compared to age matched Ndph^y/-^ mice (D-L). The digest preparations confirmed capillary free avascular zones and revealed specific changes in the appearance of retinal venules. The microaneurysms were larger in central areas (H, arrowheads) than in the periphery (K, arrowheads). An example of an acellular capillary is shown in (I, arrowhead).

Moreover, as already suggested from SLO imaging (Fig 2D), retinal digest preparations confirmed that the entire superficial plexus including the far periphery was covered by microaneurysm-like lesions (Fig 4D and 4F) with higher numbers in the central (Fig 4E and 4G) than in the peripheral area (Fig 4F and 4J). Additionally, these vascular malformations appeared larger in the central retina (Fig 4H, arrowheads) than in the more peripheral areas (Fig 4K, arrowheads).

**Fig 5:**
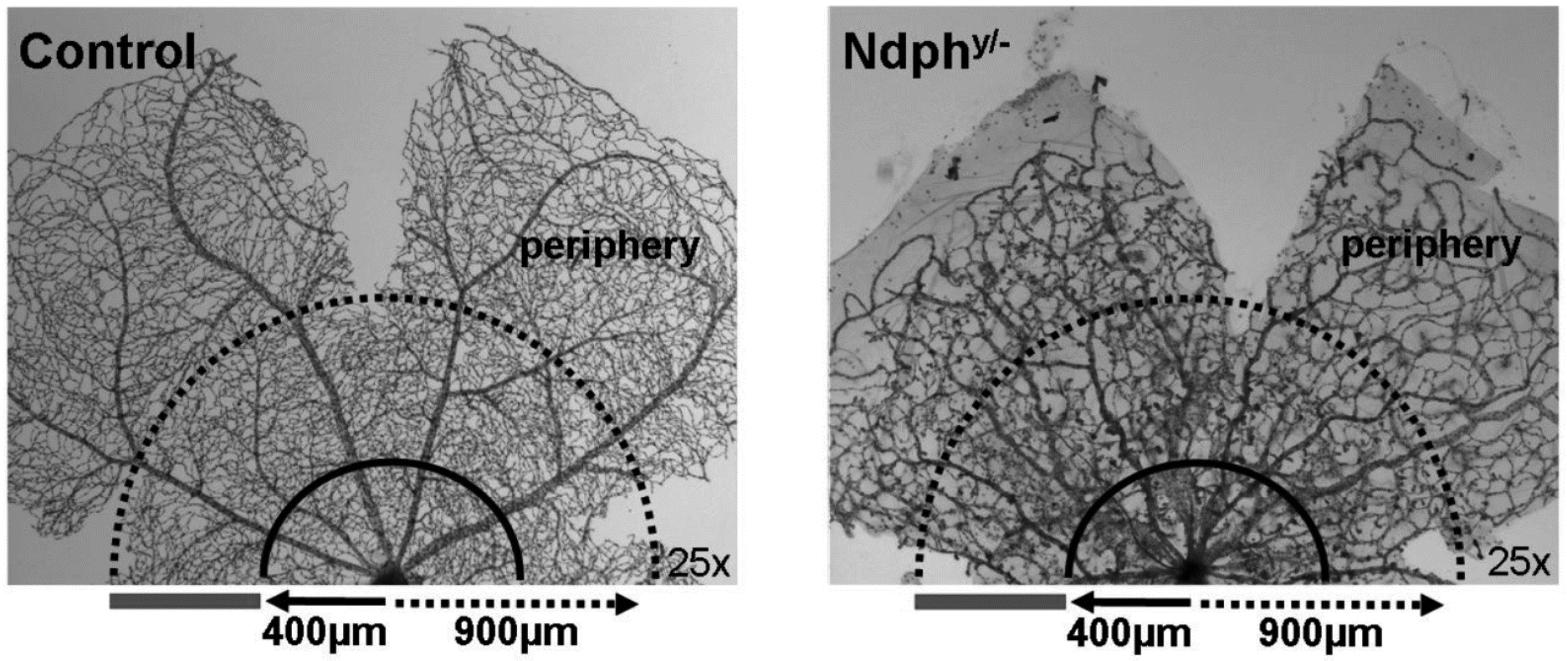
Quantification of retinal morphometric characteristics. The diameter of retinal arterioles and venules was measured at a distance of 400 μm of the optic nerve head whereas that of capillaries was assessed in the mid-central retina (400–900 μm) on PAS-stained retinal digestions. The number and the diameter of the microaneurysms were determined on arterioles and on venules in the mid-central retina within a 500 μm-thin ring distanced by 400–900 μm from the optic nerve head (grey bar).

Next we aimed to quantitate the microvascular alterations in more detail by evaluating their size and their distribution. The number and the diameter of the microaneurysm-like lesions were determined on arterioles and on venules in the mid-central retina within a 500 μm-thin ring (Fig 5) distanced by 400–900 μm from the optic nerve head. These analyses revealed that the microaneurysm-like lesions were more numerous on arterioles than on venules (mean value of 6 versus 4 microaneurysms on arterioles and venules, respectively) (Fig 3D). Similarly, the mean width of the microaneurysms in the central retina was significantly increased compared to periphery (eccentricity of more than 900 μm from the optic nerve head) (Fig 3E). Furthermore, magnifications of digest preparations as well as *in vivo* angiography revealed that large avascular areas without capillaries were not only confined to the far periphery (Fig 1B) but were present throughout the entire retina, even in the central part (Fig 3F, asterisks and Fig 4G and 4H) and close to the optical disc (Fig 3G, asterisks).

Moreover, PAS staining of retinal digest preparations allow the detection of vascular mural cells, with endothelial cells appearing lighter in staining and more elongated compared to the darker and more roundish appearance of the pericytes [17]. Thus, according to staining and shape mainly pericytes could be identified in the microaneurysm-like structures (Fig 3F, inset). Generally, in *Ndph*^*y/-*^ mice pericytes and endothelial cells were irregularly distributed and predominantly arranged in cellular clusters (Fig 3F) compared to the almost uniform distribution of vascular wall cells in the retina of WT mice (Fig 4C).

### Functional hypoxia

**Fig 6:**
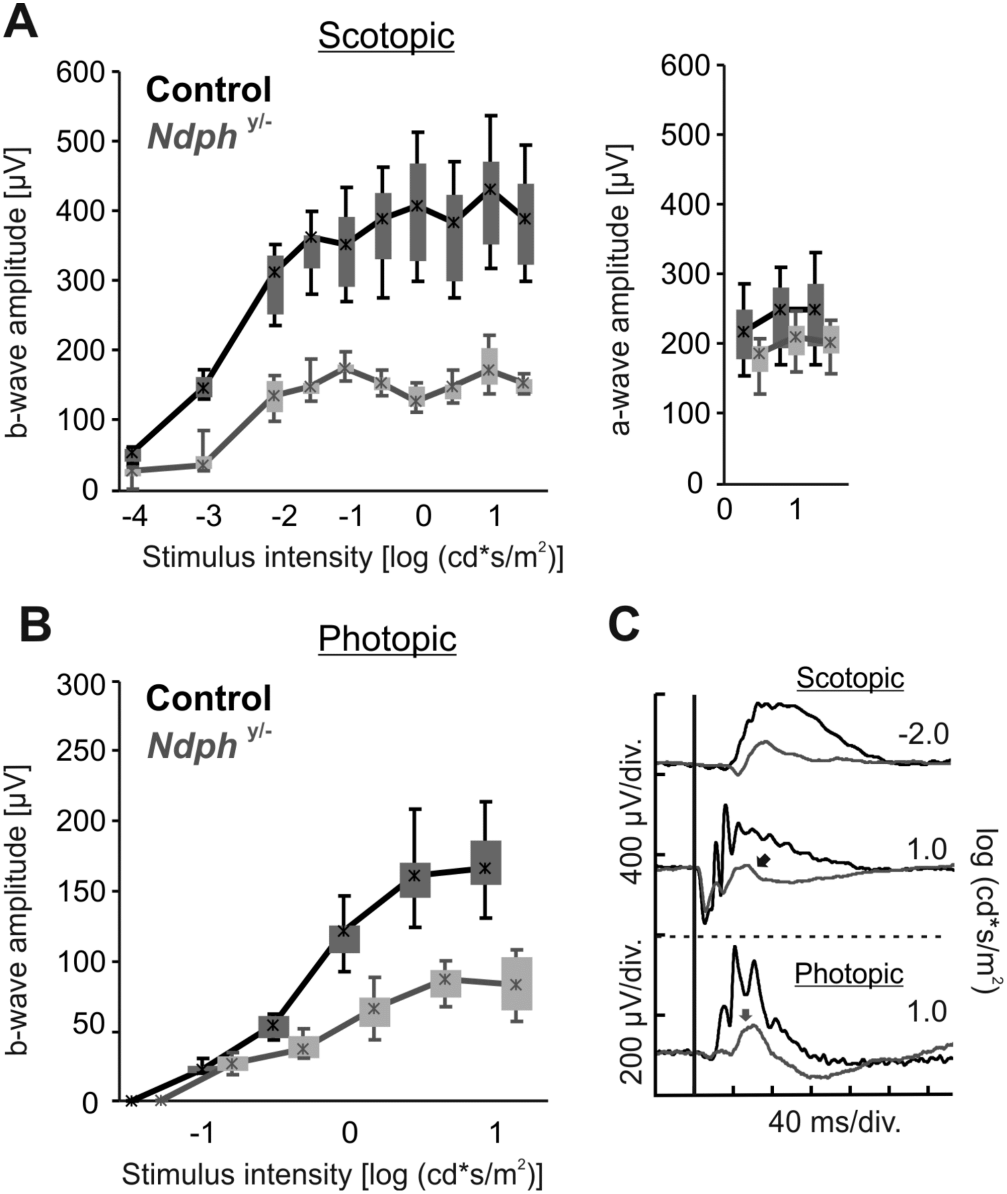
Functional assessment of *Ndph*^*y/-*^ and corresponding control mice based on ERG at 2 months. Quantitative evaluation (box-and-whisker plot) of the scotopic (A, left) and photopic (B) b-wave amplitude data for *Ndph*^*y/-*^ mice (grey) and controls (black). Additional quantification of scotopic a-wave amplitude (A, right). Boxes indicate 25% and 75% quartiles, whiskers 5% and 95% quantiles and the asterisks the median of the data. (C) Selected single flash ERG traces for the scotopic -2.0 log (cd*s/m2) stimulus intensity, representing pure rod system response (top) and the scotopic 1.0 log (cd*s/m2) stimulus intensity (center; mixed rod-cone system response) and photopic 1.0 log (cd*s/m2) stimulus intensity (bottom; cone system response). The scotopic and photopic b-wave amplitudes were significantly reduced in *Ndph*^*y/-*^ mice when compared to control animals. Representative single ERG traces showed a large reduction of b-wave amplitude leading to a “negative ERG” in scotopic conditions in *Ndph*^*y/-*^ mice and making a lack of oxygen very probable to be the source of the functional defects. In addition, a complete loss of oscillatory potentials in both scotopic and photopic conditions was observed.

Previously, functional hypoxia had been observed due to the lack of vascularization in the inner retina [12] of young *Ndph*^*y/-*^ mice during retinal development. In the mature retina, however, despite the ongoing vascular remodelling, the deeper vascular layers have still not developed at 2 months of age (Fig 2), also the retinal periphery was still not completely vascularized (Fig 1) with the result of persisting fetal vasculature in the anterior part of the eye (Fig 1). Therefore, to assess whether the vascular pathology still affects the function of retinal neural cells, functional studies based on full-field electroretinography (ERG) that allow the assessment of both, dark adapted (scotopic) rod-dominated responses and light adapted (photopic) cone-driven responses [18] have been performed in adult *Ndph*^*y/-*^ mice and age matched control animals. The analyses under scotopic and photopic conditions in control and *Ndph*^*y/-*^ mice (Fig 6) revealed a considerable reduction of about 2.5-fold of the b-wave amplitude of mutant mice compared to control animals under scotopic (Fig 6A, left) as well as photopic (Fig 6B) conditions suggesting functional defects in both, rod and cone system. Moreover, a detailed view of representative ERG recordings of 2-months-old *Ndph*^*y/-*^ mice demonstrated a large reduction of the b-wave amplitude resulting in a “negative ERG” in scotopic conditions (Fig 6C, black arrow). Another very clear difference between the ERG responses of control and mutant animals was the almost complete loss of oscillatory potentials (Fig 6C, grey arrow). Analysis of the a-wave amplitudes of *Ndph*^*y/-*^ mice revealed that they were in the normal range (between 5%-95%) of the control data (Fig 6A, right). Taken together, the strongly diminished b-wave and the almost complete loss of oscillatory potentials indicated to functional impairments of the inner retina and suggested hypoxic conditions also in the adult retina. Moreover, the comparison of the ERG responses at p21 [12] with the ERG traces of the adult *Ndph*^*y/-*^ mice (Fig 6) revealed that the functional hypoxia increased over time.

### Consequences of long-term Norrin deficiency on vascular function and integrity

**Fig 7:**
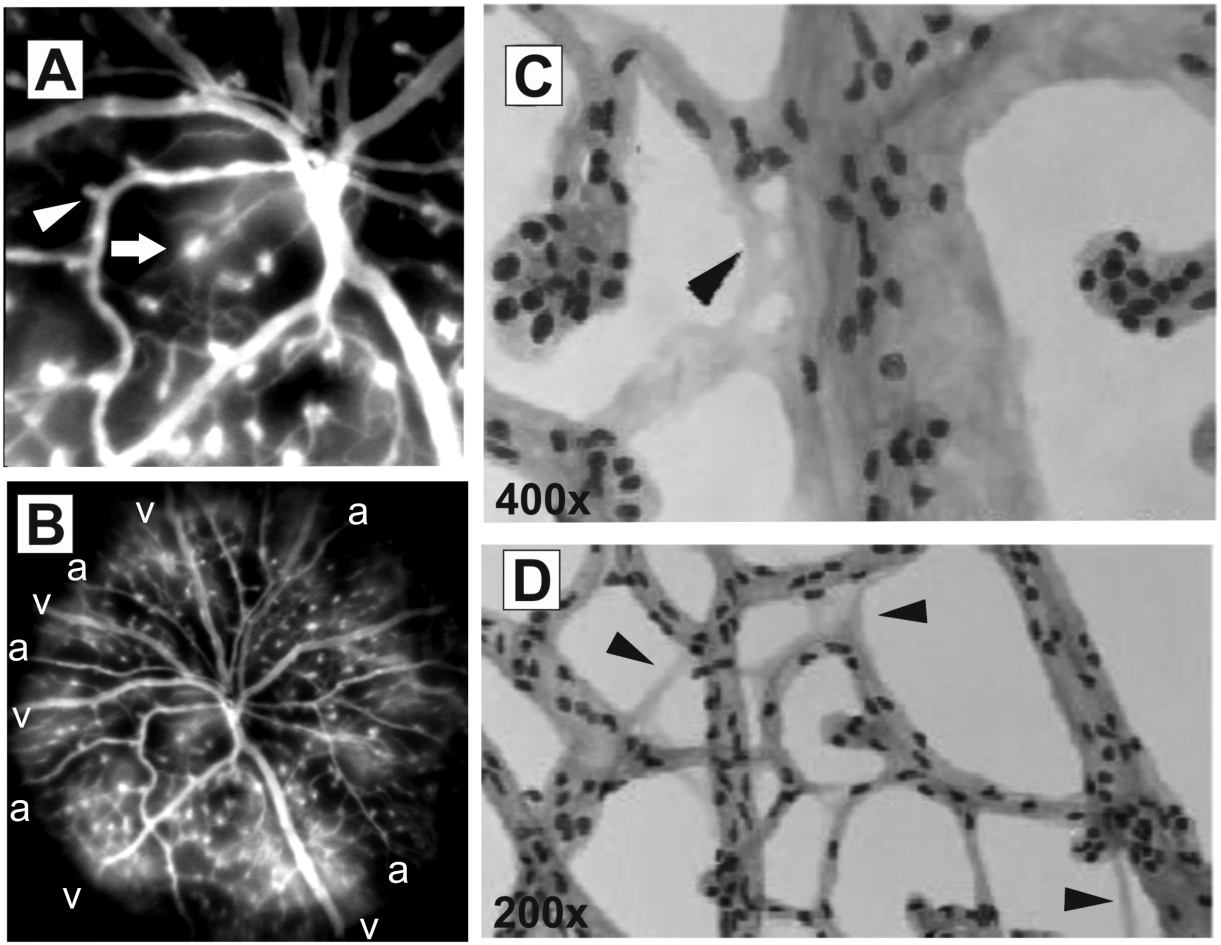
Effects of long-term Norrin deficiency on vascular function and integrity. (A) *In vivo* FL angiography detected severe leakiness of a particular subset of microaneurysm-like structures (arrow), whereas other microangiopathies presented normal vascular integrity (arrowhead). (B) SLO overview revealed bright clouds of accumulating FL around the veins (v) whereas the surroundings of arteries (a) appeared dark. Surprisingly, several acellular capillaries could be observed, both in the central region (C, arrowhead) as well as in the periphery of the retina (D, arrowheads).

Since hypoxia triggers VEGF expression that in turn leads to destabilization of the vasculature we performed *in vivo* angiography with FL that—by leaking very rapidly from fenestrated vessels—specifically allows the analysis of the vascular integrity [16]. In fact, we observed substantial extravasation of FL from microaneurysm-like lesions that indicated to fenestrated vasculature (Fig 7A, arrow). Surprisingly, not all of the vascular malformations appeared leaky, also microangiopathies that were obviously not fenestrated were observed (Fig 7A, arrowhead). The overview mode in SLO imaging actually revealed an uneven distribution of FL (Fig 7B), in particular, the bright clouds of accumulating FL were specifically detected surrounding the veins (Fig 7B, v) whereas the areas close to the arteries appeared dark (Fig 7B, a). Probably, the higher oxygen partial pressure of the arteries prevented tissue hypoxia in these regions. Whereas in the areas of the veins the oxygen supply was not sufficient resulting in local hypoxia which in turn induces the loss of vessel integrity and thus the diffusion of FL from the fenestrated vessels of the microangiopathies at these sites.

Moreover, we observed that the retinal hypoxia did not only influence the overall vessel homeostasis and integrity but also the cellular constituents of the capillaries. Very surprisingly, we could detect several acellular capillaries in the Norrin deficient retina of 2 months old mutant animals (Fig 7C and 7D). In both, the central region of the retina (Fig 7C, arrowhead) and in the periphery (Fig 7D, arrowheads), capillaries devoid of vascular wall cells were observed. Acellular capillaries are described as a consequence of pericyte drop out with subsequent loss of endothelial cells [20] and have been observed as an early morphological alteration in the diabetic retina [20]. However, this phenomenon is also known as a response to ischemia and injury in the brain [21, 22].

## Discussion

The present work provides comprehensive morphological and functional data on the long-term consequences of Norrin deficiency in the retina of adult *Ndph*^*y/-*^ mice. Detailed morphological analyses of the retinal vasculature demonstrated constant remodelling that not only affected the large retinal vessels but also developed microangiopathies along with extensive vascular fenestration. According to the pathological vascular morphology observed, functional studies revealed a considerable reduction of photoreceptor function.

In particular, some characteristics of the vascular phenotype of 2-months-old *Ndph*^*y/-*^ mice resembled that seen on the same animals at 21 days of age [12] with namely, an incomplete outgrowth of the superficial capillary network and a lack of development of the deeper capillary networks (Figs 1 and 2). These initial vascular abnormalities developed to further morphological modifications indicating to ongoing remodelling in the adult retina. Whereas the superficial retinal vascular plexus covered only 75% of the retinal surface at postnatal day 21, their percentage coverage was of 85% at 2 months of age (Fig 1B and 1C), suggesting that its development continued after postnatal day 21 probably as an attempt to compensate partially for the primary defects in angiogenic sprouting.

In addition, we observed further structural alterations in retinal vessels of adult *Ndph*^*y/-*^ mice, as indicated by the higher diameter and subsequently the increased volume of retinal venules and capillaries (Fig 3B and 3C). A similar vascular response has already been observed in different organs and tissues of animals submitted to chronic hypoxia [23, 24], and particularly in the superficial vascular bed of adult retina [24]. The observed changes in vascular diameter were limited to retinal venules and retinal capillaries without affecting the retinal arterioles in *Ndph*^*y*/-^mice (Fig 3A–3C). This at first seems surprising since it is generally thought that chronic hypoxia induces a compensatory increase of either arteries in various systems including the carotid body [25, 26], or both arteries and veins in the retina [27–29]. A potential explanation would be based on the manner the retinal vasculature develops during early life. The deeper capillary layers of the retinal vasculature are known to occur when the superficial primary plexus reaches the margins of the retina [30]. At that time, angiogenic sprouts are formed from veins, venules and capillaries near veins (but not arteries) and penetrate the retina perpendicularly to the superficial primary plexus.

We here demonstrate that in 2-months-old *Ndph*^*y/-*^ mice the retinal vessels were confined at the level of retinal nerve fibre layer and that no deeper plexuses were present (Fig 2). Since these lacking vascular networks would have developed from veins, venules and capillaries near veins, one can hypothesize a specific adaptive response of these entities. Their specific increase in size (Fig 3B and 3C) would then represent a secondary response to the lack of development of the deeper vascular layers in *Ndph*^*y/-*^ mice. Furthermore, the preferential distribution of the microaneurysms on arterioles and their dissimilar sizes (Fig 3D and 3E) could also be explained by the vascular particularities of the adult Norrin deficient retina. The reduced outgrowth of retinal superficial capillaries together with the absence of the secondary and tertiary arborisations of retinal blood vessels very likely have generated a 3-dimensional gradient of oxygen decreasing from retinal centre to periphery and from the retinal surface to deep layers. Our results clearly indicated that the microaneurysms in the retina of *Ndph*^*y/-*^ mice were larger and preferentially positioned at places where oxygen concentration may be higher, namely on arterioles and close to retinal centre (Figs 3 and 4).

As it could be suggested from the lack of vascularization of the inner retina and the persistent vascular abnormalities observed, ERG analyses revealed still functional hypoxia in the adult *Ndph*^*y/-*^ retina (Fig 6). In particular, the typical hypoxia-dependent loss of function could be observed (Fig 6). The changes in ERG waveforms in *Ndph*^*y/-*^ mice closely matched those measured during retinal but not choroidal hypoxia [31]. The almost complete loss of oscillatory potentials together with the “negative-ERG” are typical characteristics of this phenomenon (Fig 6) [32]. Without Norrin and because of the lack of a well-developed capillary bed, the retinal neuronal cells have a diminished ability to acquire nutrients and gases from the vascular system. This is particularly true for the cells from the inner retina generating the b-wave component of ERG and having oxygen supply through the retinal vasculature. Since photoreceptor cells can uptake oxygen and nutrients from the choroidal vasculature through the retinal pigment epithelium, the a-wave was not affected by hypoxia (Fig 6). Thus, the considerably lowered b-wave indicated to hypoxic conditions in the mature inner retina of 2-months-old *Ndph*^*y/-*^ mice.

A rather unexpected finding was the presence of acellular capillaries (Fig 7C and 7D). Acellular capillaries are a result of pericyte dropout that can be observed in the diabetic retina [20] and brain injuries [21, 22], but can as well be induced in specific mouse models e.g. in the platelet-derived growth factor (PDGF) knockout mice [33] or in the mouse model of inducible mural cell loss [34]. Since diabetic retinopathy is a metabolic disease and Norrin pathology is caused by a developmental defect, this at first seems surprising, but what these retinal pathologies both have in common is the presence of vascular alterations and the development of microangiopathies. In diabetic retinopathy a key regulator of vascular integrity is the angiopoietin-receptor tyrosine kinase Tie-2 system [2]. In particular, pericyte loss is triggered by the upregulation of angiopoietin-2 (Ang2) which is influenced by hyperglycemic conditions in the diabetic retina; but can as well be induced by hypoxic conditions [2]. As demonstrated, in the adult *Ndph*^*y/-*^ retina functional hypoxia could still be observed (Fig 6), thus Ang2-dependent pericyte dropout could very well be induced by persistent oxygen deficiency. Yet, it remains to be elucidated if in the Norrin deficient retina pericyte loss was triggered by the Ang-2/Tie-2 system [35] or rather by apoptosis and destructive signalling pathways [36]. In diabetic retinopathy pericyte loss precedes the development of microangiopathies [2, 37], however, this phenomenon seemed to be a secondary response in the adult Norrin depleted retina. In young *Ndph*^*y/-*^ mice retinal microangiopathies could be observed but with unaffected pericyte coverage [12], thus the pericyte drop out in the adult Norrin deficient retina must have developed in later stages perhaps as a response to persistent oxidative stress.

Generally, retinal hypoxia results in elevated levels of hypoxia-inducible factor-1 (HIF-1) which stimulates expression of VEGF and other hypoxia-regulated gene products [38] which could also be observed in the Norrin depleted retina [12]. Enhanced VEGF expression is known to induce increased permeability of endothelial cells leading to fenestrated vessels [25]. As we have shown previously [16], FL angiography allows the precise detection of fenestrated vasculature *in vivo*. In this respect, in the retina of adult *Ndph*^*y/-*^ mice we could observe severe leakiness of the microaneurysm-like lesions as indicated by substantial extravasation of fluorescein surrounding these vascular malformations (Fig 7A, magnification; 7B, overview). But not all of these structures were fenestrated. In particular, release of FL could only be observed in the microangiopathies emerging from veins (Fig 7B, v) where the oxygen partial pressure is lower. This clearly indicates to adaptive mechanisms to enhance the oxygen supply to the surrounding tissue. Which is not unlikely since a similar mechanism of oxygen distribution could be observed in the avascular retina of avian eyes. Here, the oxygen is supplied by a specific structure, called pecten oculi, characterized by manifold pleads that consist of highly fenestrated blood vessels. Oxygen is then supplied to the retina by diffusion from the blood vessels [39]. Based on this mechanism it could be suggested that the hypoxic retina in *Ndph*^*y/-*^ mice might also benefit from the fenestrated microangiopathy-like vascular abnormalities.

Taken together, the main focus of this study was the comprehensive analysis of the impact of Norrin deficiency on the adult retina. In particular, we have demonstrated that Norrin signalling is essential for vascular morphology and homeostasis also in the mature retina. The remodelling observed resulted in severe vascular alterations and the development of microangiopathies with extensive vascular fenestration, which, however, could not compensate for the lack of deeper capillary layers. As a consequence, functional hypoxia was still present. Thus, in the adult Norrin deficient retina the primary defects due to the loss of Norrin signalling developed into secondary alterations triggered by the constant oxidative stress.
